# 19 Inpatient Complications and Outcomes for Burn Patients Admitted with Opioid-Positive Drug Screens

**DOI:** 10.1093/jbcr/iraf019.019

**Published:** 2025-04-01

**Authors:** Arman Fijany, Emily Swafford, Tyler Murphy, Jordan Garcia, Punit Vyas, Robel Beyene, Stephen Gondek, Anne Wagner, Jessica Ballou, Elizabeth Slater

**Affiliations:** Vanderbilt University Medical Center; Vanderbilt Burn Center; Vanderbilt Burn Center; Vanderbilt University Medical Center; Vanderbilt University Medical Center; Vanderbilt University Medical Center; Vanderbilt University Medical Center; Vanderbilt University Medical Center; Vanderbilt University Medical Center; Vanderbilt University Medical Center

## Abstract

**Introduction:**

Drug intoxication and abuse can complicate the treatment and management of burn patients, particularly those who present with a history of opioid abuse. The severe pain associated with burn injuries makes the inpatient management of these patients challenging. While single-institution studies have described the effects of opioid use history on outcomes, there has yet to be a multi-institution database study on inpatient complications.

**Methods:**

The Python language was used to examine the American Burn Association (ABA) Noncommercial Burn Research Dataset for burn center admissions from 2012-2021 with positive drug screen results for an opioid. Within the dataset, this included any positive results for oxycodone, opiates, or opioids. The primary outcomes studied was mortality; secondary outcomes included ICU admission, respiratory failure, pneumonia, and hospital length of stay (LOS). Tertiary outcomes included renal failure, unplanned intubation, arrhythmia, cellulitis, systemic sepsis, and renal failure. Ordinary Least Squares (OLS) regression was used to describe hospital LOS. Multivariate logistic regression analysis described associations between opioid positivity and primary and secondary outcomes. All regression analyses included the following covariates: age, sex, TBSA%, and inhalation injury. The chi-square test was used to calculate odds ratios (OR) for tertiary outcomes. Data analysis was done with PyCharm 3.1 software using pandas, NumPy, and SciPy.Stats modules.

**Results:**

The ABA Noncommercial Burn Research Data Set included 51,238 drug screens from 2012-2021, of which 16,201 (31.6%) were positive for opioids. Patients with an opioid-positive drug screen were significantly more likely to be female (p < 0.005), significantly less likely to be homeless (p < 0.0001), and significantly more likely to have a scald (p < 0.0001), contact (p < 0.0001), or chemical injury (p < 0.0001). The rate of cellulitis (OR = 1.87, p < 0.0001) and systemic sepsis (OR = 1.23, p < 0.005) were significantly higher in admissions presenting with opioid positivity. After adjusting for covariates, patients with opioid positivity had a significantly higher risk of respiratory failure (OR = 1.37, p < 0.005) compared to other patients who did not test positive for opioids.

**Conclusions:**

This study represents the most extensive retrospective, multi-institutional analysis examining opioid positivity in burn center admissions. After adjusting for critical confounding factors such as age, sex, TBSA%, and inhalation injuries, opioid-positive patients were found to have significantly higher rates of respiratory failure.

**Applicability of Research to Practice:**

Our findings suggest that opioid tolerance and excessive pain management requirements with burn injuries may contribute to this finding, stressing the importance of opioid alternatives and increased clinical vigilance when treating this patient population.

**Funding for the Study:**

N/A

